# Life-threatening lower gastrointestinal bleeding after routine anorectal procedures: a case series and management implications

**DOI:** 10.1186/s12876-026-04769-7

**Published:** 2026-03-28

**Authors:** Xiangyi Yin, Xu Zhang, Ying Li

**Affiliations:** 1 Department of Proctology, Yueyang Hospital of Integrated Traditional Chinese and Western Medicine, Shanghai University of Traditional Chinese Medicine, Shanghai, China; 2 Shanghai University of Traditional Chinese Medicine, Shanghai, China

**Keywords:** Lower gastrointestinal bleeding, Enema, Case series, Colonoscopy, Anorectal invasive procedures

## Abstract

**Background:**

This case series characterizes enema- and catheter-induced rectal injuries as an underrecognized iatrogenic cause of life-threatening lower gastrointestinal bleeding (LGIB). By focusing on elderly, high-risk patients, we highlight the diagnostic complexities and management strategies associated with these routine yet potentially catastrophic anorectal procedures.

**Methods:**

We analyzed three cases of high-risk elderly patients who experienced acute, massive hemorrhage following routine anorectal procedures, specifically glycerin enemas and anorectal manometry. To contextualize these findings, we performed a narrative literature review of the PubMed and Web of Science databases to identify recurring clinical characteristics and predisposing factors associated with these iatrogenic rectal injuries.

**Results:**

All three elderly patients with significant comorbidities experienced sudden, painless, massive hematochezia following routine anorectal procedures. Standard anoscopy failed to localize the bleeding source due to obscuration by extensive blood clots. However, emergency colonoscopy without bowel preparation successfully identified and treated bleeding foci in the distal rectum (3–4 cm from the anal verge). Clinically, the patients demonstrated profound hemoglobin drops ranging from 10 to 80 g/L (nadir: 59 g/L) and severe hemodynamic instability (lowest blood pressure [BP]: 89/56 mmHg; Heart rate[HR]: 120 bpm). Two patients required massive blood transfusions, receiving up to 13 units of red blood cells and 1,350 mL of fresh frozen plasma to maintain stability. Notably, all three patients required Intensive care unit (ICU) admission for intensive monitoring and resuscitation. Common risk factors identified included advanced age, prolonged bed rest, and underlying coagulopathy.

**Conclusion:**

In frail, bedridden patients, even routine anorectal procedures such as enema administration or catheterization can trigger catastrophic, life-threatening hemorrhage. While instrumentation is the primary trigger, clinical outcomes depend on recognizing potential mucosal vulnerability. The key to survival lies in early risk stratification, maintaining a high index of clinical suspicion regarding recent procedural history, and proceeding directly to emergency colonoscopy to identify and treat hidden bleeding sources in the distal rectum. Such vigilance is not merely best practice—it is life-saving and should be formally integrated into clinical guidelines to enhance safety for high-risk populations.

## Introduction

Gastrointestinal (GI) bleeding is a critical medical emergency with a mortality rate ranging from 2% to 10% [1]. LGIB accounts for 20%–30% of these cases [2]. Previous studies identify advanced age, hemodynamic instability, and elevated serum creatinine as key predictors of poor prognosis [3, 4]. Although many LGIB cases related to benign anorectal conditions are self-limiting, rapid or extensive hemorrhage can progress to life-threatening hypovolemic shock.

The most common etiologies of LGIB include diverticulosis, malignancy, and inflammatory bowel disease [5, 6]. In contrast, iatrogenic rectal injury following routine anorectal procedures—such as enema administration or catheterization—is an underrecognized cause of massive hemorrhage.When performed correctly, this medical procedure is generally perceived as simple; however, its safety profile in high-risk populations requires careful scrutiny. Far from being strictly ‘safe’ or ‘uncommon,’ iatrogenic perforation occurs in approximately 0.02–0.04% of cases, with the risk significantly increasing in elderly patients due to decreased bowel wall tensile strength [7].Recent literature indicates that 30-day mortality after enemas for acute constipation can reach 3.9% in the elderly, primarily resulting from perforation and sepsis [8].These catastrophic complications arise through three primary mechanisms: mechanical trauma, hydrostatic pressure, and chemical necrosis (notably the ‘phosphate problem’) [9–11].Reflecting these risks, recent clinical practice guidelines, such as European Society for Medical Oncology(ESMO) 2018, strongly advise against the use of sodium phosphate enemas in patients over the age of 65 or those with any degree of renal impairment [12, 13]. While these procedures are often performed routinely, the specific clinical challenge of delayed, massive, and painless bleeding in high-risk patients remains poorly recognized and is not sufficiently highlighted in major clinical reports.

Crucially, a significant diagnostic gap exists in current practice. Guidelines from the British Society of Gastroenterology (BSG) and the American College of Gastroenterology (ACG) focus on general risk stratification but do not emphasize obtaining a history of recent anorectal procedures [14, 15]. Furthermore, the detection rate of bleeding sources in the distal rectum is often low when visualization is obscured by blood clots during standard anoscopy. Because iatrogenic injury at higher rectal sites may not manifest immediately, these cases are frequently prone to misdiagnosis or delayed intervention [16].

Whether these life-threatening events are truly rare or simply underreported remains unclear, a systematic search of public databases, such as PubMed and Web of Science, reveals that such critical incidents have not been sufficiently reported, and their specific clinical characteristics and underlying risk factors remain inadequately characterized. Therefore, we present three cases of life-threatening LGIB following routine anorectal procedures to shed light on this “hidden danger,” analyze shared risk factors, and discuss management implications to enhance clinical safety.

## Method

We retrospectively reviewed the medical records of three patients hospitalized at Shanghai Yueyang Integrated Traditional Chinese Medicine and Western Medicine Hospital between January 2025 and December 2025. These patients were transferred to the ICU due to LGIB induced by routine anorectal procedures, and were identified through urgent proctology consultations requested by the ICU.Cases were included based on the following criteria: (1) occurrence of massive, painless hematochezia following an iatrogenic intervention (e.g., enema or catheterization); (2) clinical severity defined by a significant hemoglobin drop (> 30 g/L), requirement for blood transfusion, or hemodynamic instability; and (3) bleeding localized to the distal rectum via endoscopy. We systematically extracted data from the electronic medical record system, including demographics, comorbidities, procedural details, laboratory findings, and therapeutic outcomes. To contextualize these findings, a narrative literature review of PubMed and Web of Science was performed to identify recurring risk factors in similar iatrogenic cases.

## Case presentation

### Case1

Our first case is a 79-year-old man who was already hospitalized for resection of a malignant laryngeal tumor. His medical history was notable for hypertension and a remote episode of hemorrhoidal bleeding three years earlier. He had not used nonsteroidal anti-inflammatory drugs (NSAIDs) or anticoagulants in the month before the event. Because of a temporary postoperative tracheostomy, he remained supine for approximately 20 days and developed constipation with abdominal distension and reduced bowel sounds. Abdominal computed tomography (CT) excluded bowel obstruction. To facilitate defecation, glycerin enemas were administered every three days.

Several hours after the last enema, he passed approximately 100 mL of fresh blood mixed with stool without anal pain. Given his history of hemorrhoidal bleeding, the surgical team initially suspected hemorrhoidal hemorrhage and administered intravenous batroxobin. Hours later, the situation escalated: the next day he expelled a large amount of clot and fresh blood per rectum, with tachycardia exceeding 100 beats/min.

A proctology consultation was requested. Anoscopy using a transparent open-view anoscope was essentially non-diagnostic, showing only clots, oozing blood, and stool residue with severely limited visualization. Because the bleeding continued and other sources of LGIB needed to be excluded, he was kept fasting and underwent urgent colonoscopy without bowel preparation. The scope was advanced to the hepatic flexure, yet no definite active bleeding point was identified in the colon or rectum. Meanwhile, his hemoglobin fell sharply from 139 g/L to 82 g/L, prompting emergency transfusion and transfer to the ICU for close monitoring.

The team continued to“chase”a bleeding source that remained elusive. Digital subtraction angiography (DSA) (abdominal aorta, superior mesenteric artery, celiac artery, inferior mesenteric artery, iliac arteries, and renal arteries) showed no active extravasation. A second colonoscopy, advanced to the ascending colon, revealed dark red clots extending from the hepatic flexure to the rectum but again no definite bleeding focus. Gastroscopy similarly showed no evidence of bleeding.

Despite fasting, transfusion and nutritional support, and intravenous hemostatic therapy (including octreotide acetate and sodium aminoacetate), painless rectal bleeding recurred intermittently. Over the ensuing three days, his hemoglobin declined to a nadir of 59 g/L. He developed transient hemodynamic instability, with a peak heart rate of 120 beats/min and a lowest blood pressure of 89/56 mmHg. Progressive thrombocytopenia and declining fibrinogen levels were observed, suggesting evolving consumptive coagulopathy in the setting of massive blood loss. Although formal diagnostic criteria for disseminated intravascular coagulation (DIC) were not met, the worsening coagulation profile underscored the urgency of definitively localizing and controlling the bleeding source.

**Fig. 1:**
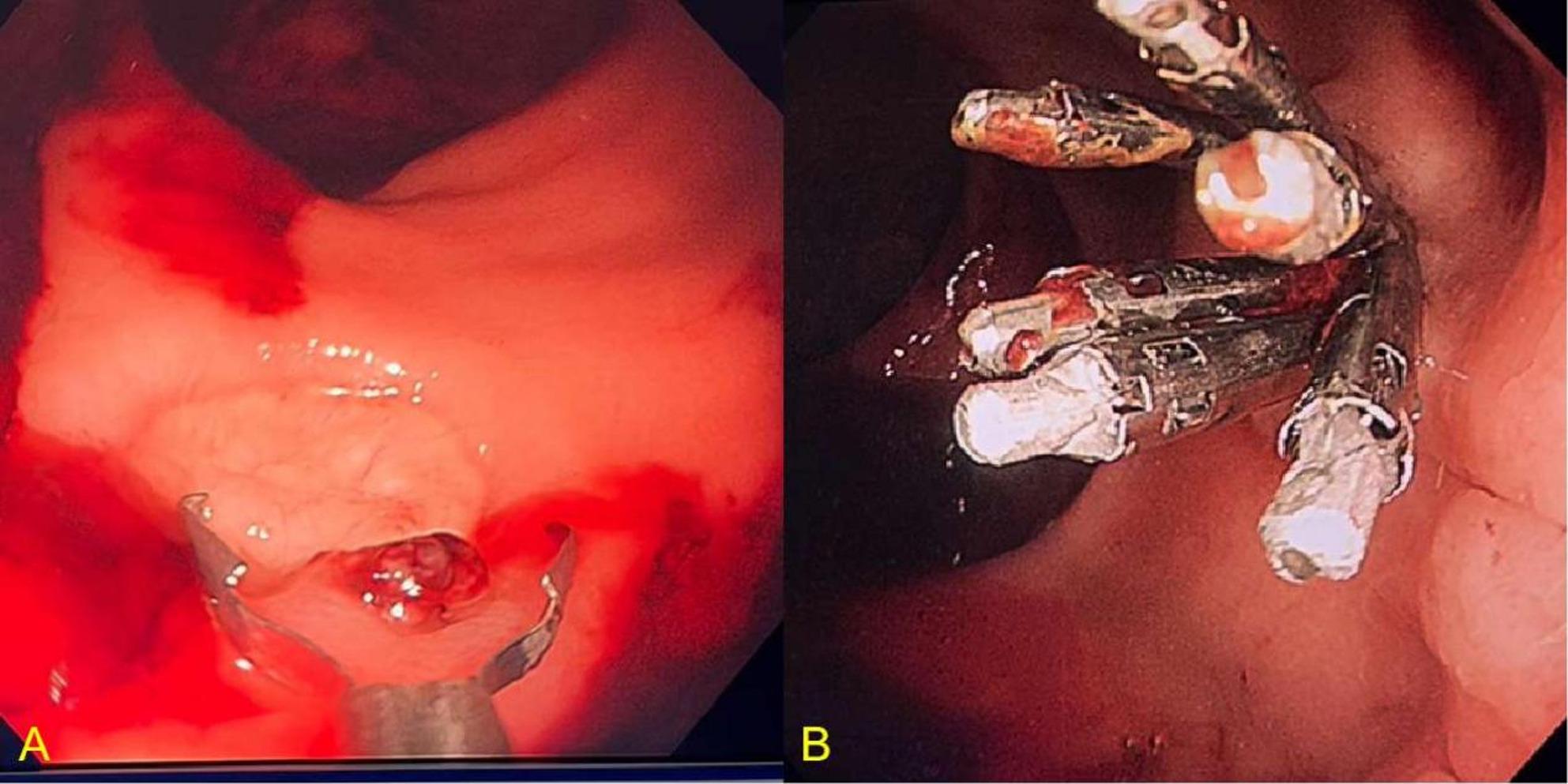
Colonoscopy findings of Case 1. **A**. Ulcerated surface and a visible vessel. **B**. Hemostasis was achieved following the application of titanium clips

A third colonoscopy was therefore performed with multiple colorectal specialists present and with particular attention directed to the distal rectum. After thorough irrigation, a small ulcer near the dentate line was finally identified, with a visible vessel on the ulcer base (Fig. 1). Although no active spurting was observed at that moment, the lesion was considered the most likely culprit. Hemostasis was achieved by applying multiple titanium clips to the visible vessel and spraying topical adrenaline.

After endoscopic therapy, only a small amount of bleeding (approximately 50 mL) occurred the next day, and no further rectal bleeding was observed. Seven days later, hemoglobin recovered to 100 g/L and platelet count and fibrinogen normalized. The patient resumed a low-residue semi-liquid diet and subsequently returned to normal bowel movements without recurrence. From the onset of bleeding to confirmation of sustained hemostasis, he received a total of 13 units of red blood cells and 600 mL of plasma.

### Case 2

Our second case is a 75-year-old man who was already critically ill. He had undergone a Billroth II gastrectomy and was admitted to the ICU for management of postoperative abdominal infection. He had been bedridden for more than 15 days. On admission, he had abdominal distension without passage of gas or stool, and feculent fluid was noted in the abdominal drainage tube. His history included prior upper gastrointestinal bleeding, but he had not used NSAIDs in the preceding month. Management included rectal gas evacuation, low-molecular-weight heparin, transfusion support, and antibiotics, and anticoagulation with low-molecular-weight heparin(3500 International units[IU] via subcutaneous injection once daily, continued throughout his hospitalization).

Three days later, his condition appeared to improve, and he passed gas for the first time. However, significant abdominal distension persisted. A coagulation screen showed an activated partial thromboplastin time Activated partial thromboplastin time.

(APTT) of 33.9 s.To relieve constipation, a routine glycerin enema was administered. Shortly afterward, he passed loose stool mixed with a small amount of dark red blood. Within three hours, the situation worsened dramatically: he passed fresh blood and clots on three occasions, and acute LGIB was diagnosed.

Rectal gas evacuation and anticoagulation were immediately discontinued, and batroxobin was administered. Despite these measures, he continued to have bloody diarrhea, with an estimated total blood loss of approximately 1250 mL. His hemoglobin dropped from 122 g/L to 92 g/L.

**Fig. 2:**
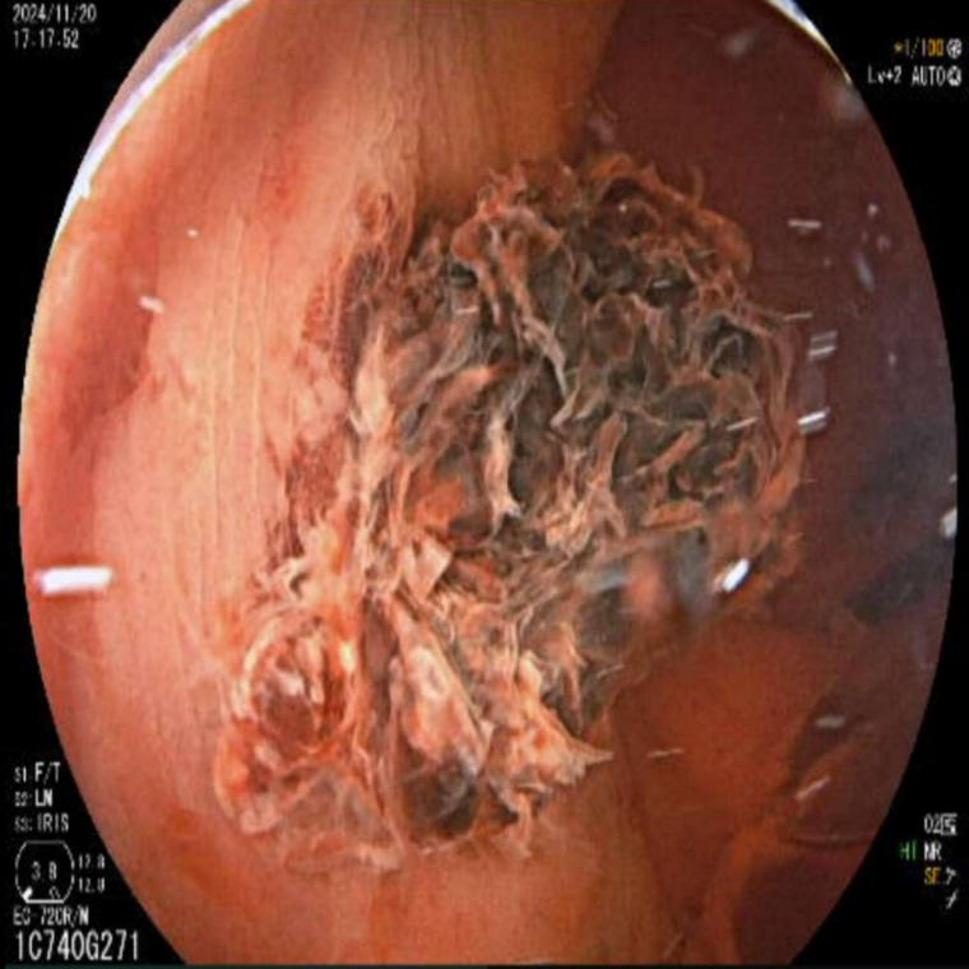
Colonoscopy findings of Case 2. Clot observed in the colon

Proctology consultation was requested. Anoscopy was attempted but proved unhelpful, as large clots obscured visualization. Given ongoing bleeding and the risk of hemodynamic deterioration in a patient with active infection, urgent colonoscopy was performed without bowel preparation. No significant lesions were found in the proximal colon. However, the rectum contained large clots and fresh blood. After repeated irrigation and suction, a firmly adherent coagulum was identified approximately 4 cm proximal to the anal verge(Fig. 2). This lesion was treated locally with adrenaline and iced saline irrigation, and no further active oozing was observed.

After endoscopic therapy, no additional episodes of fresh hematochezia occurred. Rectal retention therapy with a soft, rounded-tip enema containing adrenaline and iced saline was continued, along with transfusion support. On the following day, digital rectal examination revealed no blood. Hemoglobin gradually improved, oral intake was resumed progressively, and no rebleeding occurred. During the bleeding episode, the patient received a total of 1350 mL of plasma and 10 units of cryoprecipitate.

### Case 3

Our third case involves a 70-year-old man admitted for recurrent urinary retention of two months’ duration, scheduled for urethral metallic stent placement. His medical history was significant for hepatocellular carcinoma treated with interventional ablation. He was receiving targeted therapy and had ascites and hypoalbuminemia. He had been taking clopidogrel chronically, which was discontinued one week before admission. On admission, laboratory testing revealed thrombocytopenia (66 × 10⁹/L) and prolonged prothrombin time.

As part of preoperative evaluation, routine urodynamic testing was performed by an experienced physician. Immediately after removal of the rectal pressure catheter (8 cm, flexible rounded tip), significant rectal bleeding occurred. Anoscopy was attempted but visualization was limited by fresh blood, and the bleeding source could not be clearly identified.

Initial hemostasis was achieved with batroxobin-soaked gauze compression. However, during urination, increased intra-abdominal pressure triggered recurrent bleeding of approximately 100 mL. Irrigation with iced saline and administration of two ampoules of adrenaline were performed, after which no active bleeding was observed. The patient was kept fasting.

**Fig. 3:**
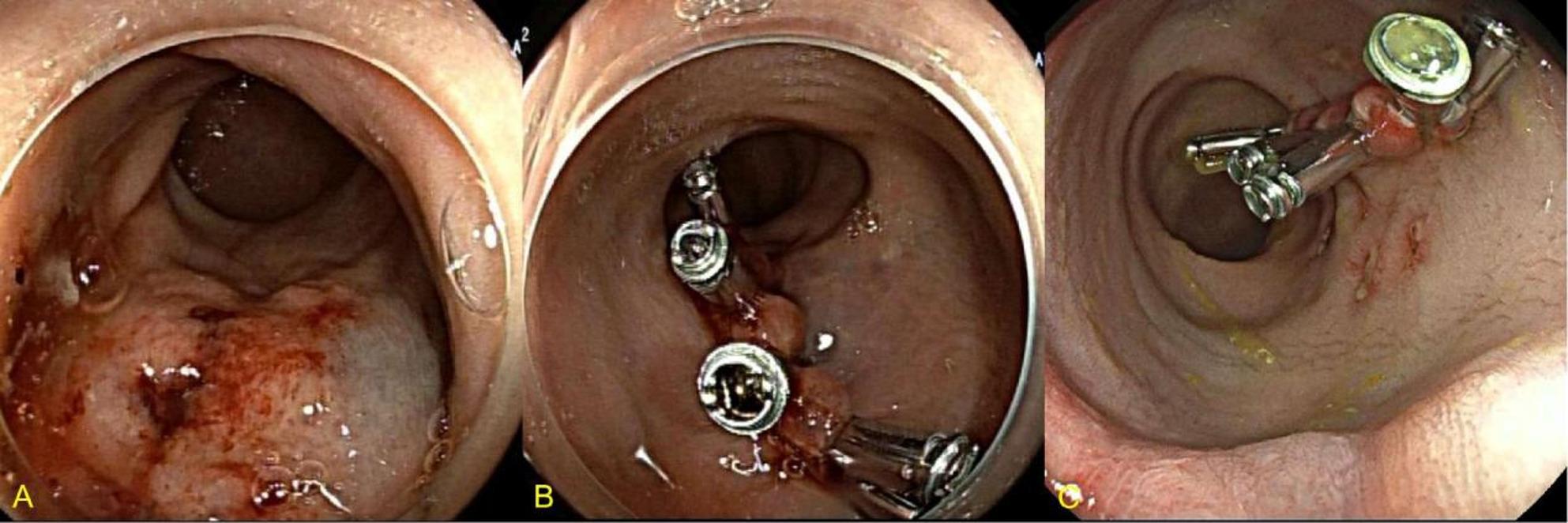
Colonoscopy findings of Case 3. **A**. Initial colonoscopy showing the ulcer. **B**. Bleeding control with titanium clips. **C**. No active bleeding or visible vessels observed during colonoscopy

Given his underlying liver malignancy, thrombocytopenia, and coagulation abnormalities, urgent colonoscopy was performed without bowel preparation. Three areas of mucosal disruption due to ulceration were identified approximately 3 cm proximal to the anal verge.Hemostasis was achieved by placement of titanium clips (Fig. 3 A, B).

Three days later, he developed a small amount of rectal bleeding after consuming a liquid diet. Repeat colonoscopy demonstrated the previously clipped sites with mild mucosal erosion but no active bleeding and no visible vessels (Fig. 3 C). After dietary adjustment to a semi-solid diet and stool control, no further bleeding occurred. He required no blood transfusion and subsequently completed the planned surgical procedure before discharge.

## Discussion

This case series presents three elderly patients who developed LGIB due to enema administration or rectal pressure catheter insertion. All patients shared the following characteristics: a history of local invasive anal procedures, bright red blood or blood mixed with clots, significant blood loss, and rapid blood flow. Active bleeding or discharge of dark blood from the rectum reappeared when abdominal pressure increased (e.g., during urination). Additionally, all three patients shared a history of prolonged bed rest and poor nutritional status, with some having malignancies or being on anticoagulant medication. The underlying pathophysiology of massive hemorrhage following routine anorectal instrumentation in our cohort remains complex and multifactorial. We propose that the interplay between mechanical stress and localized mucosal ischemia serves as the primary driver. In elderly or bedridden patients, prolonged immobility and a dependent supine position may induce “hemodynamic vulnerability” in the rectal mucosa due to venous congestion and compromised arterial perfusion [17–19]. This state is structurally and clinically analogous to Acute Hemorrhagic Rectal Ulcer (AHRU), a condition characterized by the sudden onset of painless, large-volume rectal bleeding near the dentate line [20]. While AHRU is often considered a spontaneous event linked to risk factors like hospitalization, anticoagulant use, and hypoalbuminemia [17, 21, 22]—all of which were present in our patients—our cases demonstrate that anorectal instrumentation can act as the decisive mechanical “insult” that triggers these compromised vessels. Specifically, in Case 3, the relationship between the characteristic circular ulcers of AHRU and the use of a rectal pressure catheter suggests a ‘double-hit’ mechanism. It remains uncertain whether the catheter’s localized mechanical stress served as the primary insult that initiated the ulcerative process, or as a provocative trigger that induced massive bleeding from pre-existing subclinical lesions. Regardless of the exact sequence, our findings indicate that in vulnerable patients with microvascular compromise, anorectal instrumentation can act synergistically with underlying mucosal ischemia to cause catastrophic bleeding. Thus, the clinical focus should shift from distinguishing between ‘iatrogenic’ and ‘spontaneous’ causes to recognizing instrumentation as a critical determinant of active hemorrhage in this high-risk population.

Rectal injury caused by enema administration and other medical devices—including the passage and use of rectal decompression tubes—may not result in immediate pain or rectal bleeding [23], unless the enema solution is irritating. The use of rectal decompression tubes presents an additional iatrogenic risk, as prolonged or improper placement can exert continuous localized pressure on the fragile rectal wall, potentially exacerbating mucosal ischemia and leading to disruption.This risk is particularly pronounced in patients with diabetes mellitus, as chronic microangiopathy and compromised blood flow render the anorectal mucosa fragile and highly susceptible to mechanical trauma from routine instrumentation. Glycerin enemas are among the most widely used agents for constipation due to their perceived safety and ease of administration [24]; however, they have been rarely reported to cause severe complications, such as hemolysis or renal damage, following rectal injury in diabetic patients.Most patients, particularly those with dementia, are unable to report these self-administered medical actions [25, 26]. This underscores the importance for clinicians to carefully inquire about any history of invasive anal procedures.

When rectal bleeding presents as hematochezia, clinicians typically prioritize differential diagnoses such as hemorrhoids, anal fissures, polyps, and tumors, and proceed with digital rectal examination and anoscopy. Hemostatic interventions at the anal canal and rectal distal end can be performed under anoscopic guidance. In patients with adequate coagulation function, bleeding can often be controlled effectively through the use of coagulants or iced saline enemas [27, 28]. Additionally, gauze packing is a simple and effective method, though clinicians must consider the risk of mucosal tearing. If the bleeding source is located in the middle or lower rectum, even if the bleeding point can be visualized with the anoscope, it is often not feasible to perform suturing or ligation via anoscopy, and the risk of rebleeding remains [29].

There is some disagreement in various guideline studies regarding the timing of colonoscopy intervention in patients with acute LGIB [15, 30, 31], likely due to the self-limiting nature of some LGIB cases. However, for patients with a clear medical historyand ongoing or persistent rectal bleeding, especially those in whom hemostasis cannot be achieved via anoscopy, performing endoscopy to identify the active bleeding source is essential.

Clearly, the patients in this case series urgently required early colonoscopy. This approach not only helps reduce treatment costs and shorten hospitalization [32] but also minimizes the use of valuable blood products. Another important consideration is bowel preparation. Previous studies have shown a high success rate for emergency colonoscopy without bowel preparation [33, 34]. Therefore, the lack of bowel preparation in certain LGIB patients does not constitute an obstacle to performing colonoscopy. There are many hemostatic options available during endoscopy, including adrenaline injection, titanium clips, argon plasma coagulation, and others. In this case series, patients underwent fasting and stool control after endoscopic treatment and did not experience further bleeding.

With the rapid development of multidisciplinary technologies and significant improvements in endoscopic diagnostic and therapeutic capabilities, there have been important advances in the treatment strategies for LGIB in the rectal segment. Several case reports have described the use of the Sengstaken-Blakemore tube in cases of rectal bleeding, including those secondary to enema-induced injury [9, 35], when electrocautery or clip application fails, or when the cause of bleeding is unknown [35–37]. Additionally, rectal arterial embolization (RAE) [38], a minimally invasive treatment, can be considered in a multidisciplinary context for patients who cannot tolerate bowel preparation. However, as demonstrated in Case 1, if the visible vessel is not actively bleeding, negative angiographic results may lead to treatment delays.

This case series provides several practical lessons. First, despite massive bleeding or a lack of bowel preparation, early colonoscopy remains essential, with particular attention required for the distal rectal mucosa. Second, to ensure procedural safety, patients should be positioned in the Sims’ position (lying on the left side with knees bent toward the chest). Instrumentation must be gentle, with the tip directed toward the umbilicus. Repeated procedures within a short period should be strictly avoided to prevent mucosal injury or perforation [25, 39]. Finally, clinicians must maintain a high index of suspicion and specifically inquire about any recent anorectal manipulations, such as enemas or anorectal manometry [40].

**Fig. 4:**
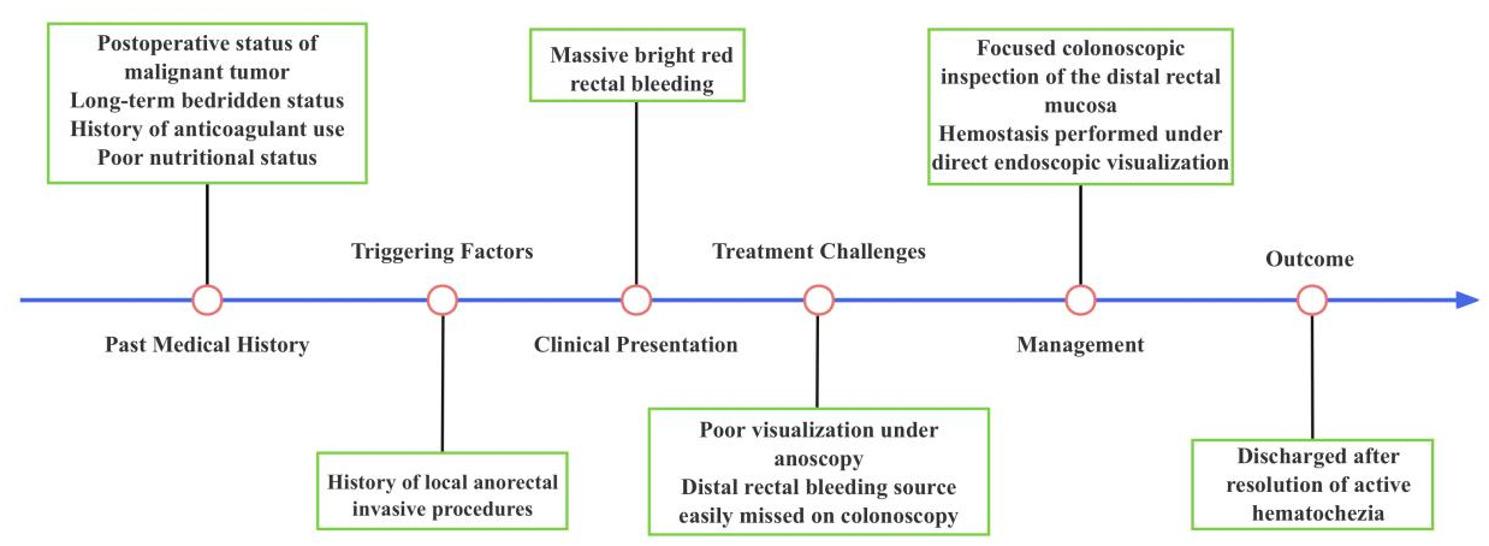
Timeline of clinical progression

The whole timeline of clinical progression is shown in Fig. 4.

## Conclusion

In conclusion, invasive anorectal procedures can trigger life-threatening LGIB in high-risk elderly or bedridden populations. While early endoscopic intervention remains the definitive strategy for diagnosis and hemostasis, prevention is paramount. Regarding clinical protocols, while current guidelines for LGIB do not ignore the necessity of history-taking, they do not explicitly emphasize recent anorectal instrumentation as a mandatory diagnostic element. We propose that future updates to these guidelines should more clearly highlight the importance of obtaining a specific history of anorectal procedures in high-risk patients to mitigate diagnostic delays and enhance patient outcomes.

## Data Availability

No datasets were generated or analysed during the current study.

## Abbreviations

LGIB: Lower Gastrointestinal Bleeding
BP: Blood Pressure
HR: Heart Rate
ICU: Intensive Care Unit
GI: Gastrointestinal
ESMO: European Society for Medical Oncology
BSG: British Society of Gastroenterology
ACG: American College of Gastroenterology
NSAIDs: Nonsteroidal Anti-inflammatory Drugs
CT: Computed Tomography
DSA: Digital Subtraction Angiography
DIC: Disseminated Intravascular Coagulation
IU: International Units
APTT: Activated Partial Thromboplastin Time
AHRU: Acute Hemorrhagic Rectal Ulcers
RAE: Rectal Arterial Embolization
AHRU: Acute Hemorrhagic Rectal Ulcers
